# The sequence and *de novo* assembly of *Takifugu bimaculatus* genome using PacBio and Hi-C technologies

**DOI:** 10.1038/s41597-019-0195-2

**Published:** 2019-09-30

**Authors:** Zhixiong Zhou, Bo Liu, Baohua Chen, Yue Shi, Fei Pu, Huaqiang Bai, Leibin Li, Peng Xu

**Affiliations:** 1 State Key Laboratory of Marine Environmental Science, College of Ocean and Earth Sciences, Xiamen University, Xiamen, 361102 China; 2 Fisheries Research Institute of Fujian, Xiamen, 361000 China; 3 State Key Laboratory of Large Yellow Croaker Breeding, Ningde Fufa Fisheries Company Limited, Ningde, 352130 China; 4 Laboratory for Marine Biology and Biotechnology, Pilot National Laboratory for Marine Science and Technology (Qingdao), Qingdao, 266071 China

**Keywords:** Genome, Sequencing, DNA sequencing, Ichthyology

## Abstract

*Takifugu bimaculatus* is a native teleost species of the southeast coast of China where it has been cultivated as an important edible fish in the last decade. Genetic breeding programs, which have been recently initiated for improving the aquaculture performance of *T*. *bimaculatus*, urgently require a high-quality reference genome to facilitate genome selection and related genetic studies. To address this need, we produced a chromosome-level reference genome of *T*. *bimaculatus* using the PacBio single molecule sequencing technique (SMRT) and High-through chromosome conformation capture (Hi-C) technologies. The genome was assembled into 2,193 contigs with a total length of 404.21 Mb and a contig N50 length of 1.31 Mb. After chromosome-level scaffolding, 22 chromosomes with a total length of 371.68 Mb were constructed. Moreover, a total of 21,117 protein-coding genes and 3,471 ncRNAs were annotated in the reference genome. The highly accurate, chromosome-level reference genome of *T*. *bimaculatus* provides an essential genome resource for not only the genome-scale selective breeding of *T*. *bimaculatus* but also the exploration of the evolutionary basis of the speciation and local adaptation of the Takifugu genus.

## Abstract (summary)

**Table:** 

Measurement(s)	whole genome sequencing assay • transcription profiling assay
Technology Type(s)	DNA sequencing • RNA sequencing
Factor Type(s)	organism part
Sample Characteristic - Organism	Takifugu bimaculatus

Machine-accessible metadata file describing the reported data: 10.6084/m9.figshare.9793001

## Background & Summary

Takifugu, belongs to Tetraodontidae in *Tetraodontiformes*, is native to estuaries and the offshore area of the northwest Pacific^1^. Despite the lethal amounts of tetrodotoxin in their bodies, Takifugu are still considered a delicacy in East Asia. Takifugu is also an established teleost model species due to its compact genome. As the first sequenced teleost genome, the genome of *Takifugu rubripes* was completely sequenced in 2002^1^. Another important Takifugu species, *Takifugu bimaculatus* (Fig. S1a), is a typically endemic species in the marginal sea from the south Yellow Sea to the South China Sea. *T*. *bimaculatus* inhabits lower latitudes and adapts to higher temperatures than *T*. *rubripes*^2^, providing an excellent model for exploring thermal adaptation and adaptive divergence in teleost fishes. In the past decade, *T*. *bimaculatus* has been widely cultured in southeast China, where the temperature is beyond the upper thermal tolerance of *T*. *rubripes*. Recently, genetic breeding programs of *T*. *bimaculatus* have been initiated, mainly aiming to improve growth rates and disease resistance under aquaculture conditions. Therefore, there is an urgent need to collect sufficient genetic materials and genome resources to facilitate genome-scale studies and selective breeding. However, a highly accurate, chromosome-level reference genome of subtropical Takifugu species is still lacking, which hinders the progress of genetic improvement and genetic studies of its thermal plasticity and adaptation at lower latitudes.

In this report, we provided a chromosome-level reference genome of *T*. *bimaculatus* using a combination of the PacBio single molecule sequencing technique (SMRT) and high-through chromosome conformation capture (Hi-C) technologies. We assembled the genome sequences into 2,193 contigs with a total length of 404.21 Mb and a contig N50 length of 1.31 Mb. After chromosome-level scaffolding, 22 scaffolds were constructed corresponding to 22 chromosomes with a total length of 371.68 Mb (92% of the total length of all contigs). Furthermore, we identified 109.92 Mb (27.20% of the assembly) of repeat content, 21,117 protein-coding genes and 3,471 ncRNAs. In addition, we also assembled a chromosome-level reference genome of *Larimichthys crocea*^3^, which is one of the top commercial marine fishery species in China, via almost the same strategy. The wo high-quality assembled genomes confirmed the stability and suitability of this strategy for marine fishes. The availability of a chromosome-level, well-annotated reference genome is essential to support basic genetic studies and will contribute to genome-scale selective breeding programs for these important maricultural species.

## Methods

### Ethics statement

The *T*. *bimaculatus* used in this work were obtained from Zhangzhou, Fujian Province, China. This work was approved by the Animal Care and Use committee at the College of Ocean and Earth Sciences, Xiamen University. All the methods used in this study were carried out following approved guidelines.

### Sample collection and nucleic acid preparation

Two healthy female *T*. *bimaculatus* was collected from an off-shore area by the Fujian Takifugu Breeding Station in Zhangzhou, Fujian Province, China (Fig. S1b); one of fish was used for SMRT and RNA sequencing, and the other fish was used for Hi-C. The muscle was collected for DNA extraction and nine different tissues (Table S1) were collected for RNA extraction. To protect the integrity of the DNA, all samples were immediately frozen in liquid nitrogen for 20 min and then stored at −80 °C. Sufficient frozen muscle tissues were lysed in SDS digestion buffer with proteinase K, and high-molecular-weight (HMW) genomic DNA (gDNA) for SMRT and Hi-C was extracted by AMPure XP beads (Beckman Coulter, High Wycombe, UK), washed with 70% alcohol and dissolved in nuclease-free water. In addition, normal-molecular-weight (NMW) gDNA for Illumina sequencing was also extracted from muscle tissues using the established method^4^. Total RNA was extracted using the TRIZOL Kit (Invitrogen, Carlsbad, CA, USA) from different tissues following the manufacturer’s protocol^5^ and mixed equally for RNA-Seq. Nucleic acid concentrations were quantified using a Qubit fluorometer (Thermo Fisher Scientific, Waltham, MA), and then checked by 1.5% agarose gel electrophoresis stained for integrity.

### Library construction and sequencing

A genome survey was performed based on Illumina short reads for estimating genome size, heterozygosity and repeat content, which provides a basic evaluation before we started the large scale whole genome sequencing. A library with a 350 bp insert size was constructed from NMW gDNA following the standard protocol provided by Illumina (San Diego, CA, USA). The library was then sequenced with a paired-end sequencing strategy using the Illumina HiSeq 2500 platform, and the read length was 2 × 150 bp. Finally, ~53.43 Gb raw data were generated. After removing the low-quality bases and paired reads with the Illumina adaptor sequence using SolexaQA++ ^6^ (version v.3.1.7.1), a total of ~53.28 Gb clean reads, were retained for the genome survey (Table 1).

**Table 1:** Summary of genome sequencing data generated with multiple sequencing technologies.

Library Type	Insert Size (bp)	Raw Data (Gb)	Clean Data (Gb)	Average Read Length (bp)	N50 Read Length (bp)	Sequencing Coverage (X)
Illumina	350	53.43	53.28	150	150	135.52
PacBio	20,000	28.97	—	7,505	12,513	73.69
Hi-C	—	46.39	46.13	150	150	117.8
RNA-Seq	—	21.35	20.95	150	150	54.3
Total	—	149.99	—	—	—	381.5

Note: Genome size of *T*. *bimaculatus* used to calculate sequencing coverage were 393.15 Mbp, which is estimated by genome survey.

For the preparation of the single-molecule real-time (SMRT) DNA template, the HMW gDNA was sheared into large fragments (10 K bp on average) by ultrasonication and then end-repaired according to the manufacturer’s instructions (Pacific Biosciences). The blunt hairpins and sequencing adaptor were ligated to the DNA fragments, DNA sequencing polymerases were bound to the SMRTbell templates. Finally, the library was quantified using a Qubit 4 Fluorometer (Invitrogen, USA). After sequencing with the PacBio SEQUEL platform at Novogene (Tianjin), a total of 3.86 Million (~28.97 Gb) long reads were generated and used for the following genome assembly. The average and N50 length of the subreads sequences were 7,505 bp and 12,513 bp, respectively. According to the genome survey, the genome size of *T*. *bimaculatus* was estimated to be 393.15 Mb; therefore, the average sequencing coverage was 73.69× (Table 1).

For Hi-C sequencing, the *Mbol* restriction enzyme was used to digest the HMW gDNA after fixing the conformation of HMW gDNA by formaldehyde, after which the 5′ overhangs were repaired with biotinylated residues. The isolated DNA was reverse-crosslinked, purified and filtered for biotin-containing fragments after blunt-end ligation *in situ*. Thereafter, the DNA was sheared into fragments by ultrasonication and subsequently repaired by T4 DNA polymerase, T4 polynucleotide kinase and Klenow DNA polymerase. Then, dATP was attached to the 3′ ends of the end-repaired DNA, and 300–500 bp fragments were retrieved by Caliper LabChip Xte (PerkinElmer, USA). The DNA concentration was quantified by a Qubit 4 Fluorometer, and the Illumina Paired-End adapters were ligated to the DNA by T4 DNA Ligase. The 12-cycle PCR products were purified by AMPureXP beads. Finally, sequencing of the Hi-C library was performed on an Illumina HiSeq 2500 platform and yielded a total of 128.64 Gb paired-end raw reads, with an average sequencing coverage of 117.80X (Table 1).

The cDNA library was prepared following the protocols of the Illumina TruSeq RNA Sample Preparation Kit (Illumina, San Diego, CA, USA) and quantitated with KAPA Library Quantification Kits. Then, sequencing of RNA-seq was performed on an Illumina HiSeq 2500 platform with a 150 bp paired-end strategy. Finally, we generated 21.35 Gb paired-end raw reads and 20.95 Gb paired-end clean reads for gene structure annotation (Table 1).

### *de novo* assembly of the *T*. *bimaculatus* genome

**Fig. 1:**
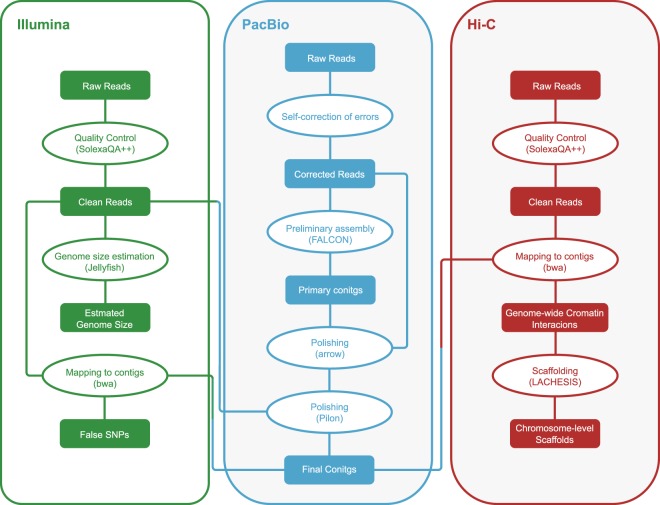
The genome assembly pipeline.

Reads from the three types of libraries were used in different assembly stages separately (Fig. 1). Illumina sequencing data, PacBio sequencing and Hi-C reads were used for the genome survey, contig assembly and chromosome-level scaffolding, respectively.

In the genome survey, paired reads with “N” sites exceeding 8 or low-quality (Q < 5) bases exceeding 60 were filtered out from the Illumina library. The pair reads containing the Illumina adaptor sequence were also filtered. Using Jellyfish^7^, the frequency of 17-mers in the Illumina clean data was calculated with a 1 bp sliding window using the established method^8^ and obeyed the theoretical Poisson distribution (Fig. S2). Finally, the proportion of heterozygosity in the *T*. *bimaculatus* genome was evaluated as 0.55%, and the genome size was estimated as 393.15 Mb, with a repeat content of 25.29% (Table S2).

Long reads generated from the PacBio SEQUEL platform were subsequently processed by a self-correction of errors using FALCON^9^. Based on the Overlap-Layout-Consensus algorithm, we detected overlaps from input reads and assembled the final String Graph^10^. Subsequently, we used the FALCON-unzip pipeline to generate phased contig sequences for further calling highly accurate consensus sequences using variantCaller in the GenomicConsensus package, which was employed as an arrow algorithm, and contigs were polished using Illumina reads by Pilon^11^. Finally, we obtained the assembled genome of *T*. *bimaculatus*, which contained including 2,193 contigs with a total length and contig N50 length of 404.21 Mb and 1.31 Mb, respectively (Table 2).

**Table 2:** Statistics of the genome assembly of *T*. *bimaculatus*.

	length		Number	
	Contig (bp)	Scaffold (bp)	Contig	Scaffold
Total	404,208,938	404,312,138	2,193	1,161
Max	8,128,173	28,865,866	—	—
Number >= 2000	—	—	2,143	1.111
N50	1.312,995	16,785,490	82	11
N60	951,152	16,217,719	117	13
N70	563,057	15,683,578	173	16
N80	220,884	13,896,868	292	19
N90	68,784	10,376,233	627	22

For chromosome-level scaffolding, we first filtered Hi-C reads with the same protocol as Illumina reads. Subsequently, we mapped the Hi-C clean reads to the *de novo* assembled contigs by using BWA^12^ (version 0.7.17) with the default parameters. We removed the reads that did not map within 500 bp of a restriction enzyme site. Using LACHESIS^13^ (version 2e27abb), we assembled chromosome-level scaffolding based on the genomic proximity signal in the Hi-C data sets. In this stage, all parameters were default except for CLUSTER_N, ORDER_MIN_N_RES_IN_SHREDS and CLUSTER_MIN_RE_SITES, which set as 22, 10 and 80, respectively. As a result, we generated 22 chromosome-level scaffolds containing 1,242 contigs (56.63% of all contigs) with a total length of 371.68 Mb (91.95% of the total length of all contigs), and the lengths of chromosomes ranged from 10.38 Mb to 28.86 Mb (Table 3).

**Table 3:** Summary of assembled 22 chromosomes of *T*. *bimaculatus*.

Chromosomes	Length (Mbp)	Number of Contigs
Chr1	28,856,866	68
Chr2	20,901,650	55
Chr3	20,839,560	60
Chr4	19,082,936	61
Chr5	18,556,983	59
Chr6	17,762,956	51
Chr7	17,385,507	47
Chr8	17,095,808	54
Chr9	17,068,765	55
Chr10	16,786,025	53
Chr11	16,785,490	54
Chr12	16,284,555	50
Chr13	16,217,719	54
Chr14	16,120,980	47
Chr15	16,059,269	50
Chr16	15,683,578	65
Chr17	14,840,516	62
Chr18	14,847,795	52
Chr19	13,896,868	51
Chr20	13,487,414	56
Chr21	12,729,218	46
Chr22	10,376,233	40
Linked Total	371,675,691	1,242
Unlinked Total	32,532,707	951
Linked Percent	91.95%	56.63%

### Repeat sequences and gene annotation

**Fig. 2:**
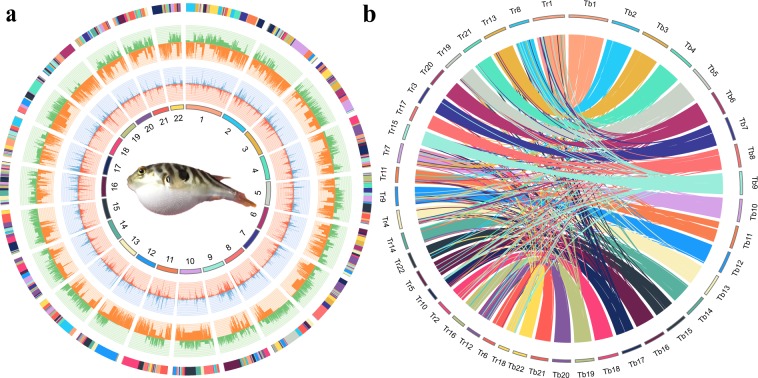
Circos plot of the reference genome of *T*. *bimaculatus* and syntenic relationship with the *T*. *rubripes* genome. (**a**) Circos plot of 22 chromosomes and the annotated genes, ncRNAs and transposable elements of *T*. *bimaculatus*. The tracks from inside to outside are 22 chromosome-level scaffolds, the positive-strand gene abundance (red), negative-strand gene abundance (blue), positive-strand TE abundance (orange), negative-strand TE abundance (green), ncRNA abundance of both strands, and contigs that comprised the scaffolds (adjacent contigs on a scaffold are shown in different colours). (**b**) Circos diagram between *T*. *bimaculatus* and *T*. *rubripes*. Each coloured arc represents a 1 Kb fragment match between two species. We re-ordered the chromosome numbers of *T*. *rubripes* for better illustration.

**Fig. 3:**
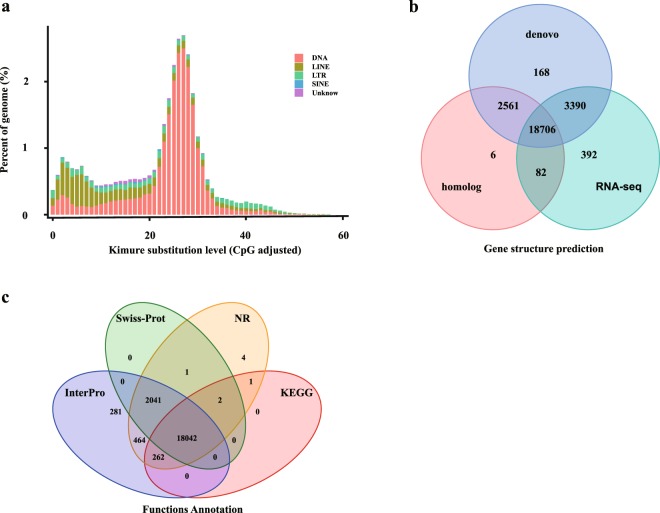
Gene and repetitive element annotations of the *T*. *bimaculatus* genome. (**a**) Divergence distribution of TEs in the *T*. *bimaculatus* genome (**b**) Venn diagram of the number of genes with structure prediction based on different strategies. (**c**) Venn diagram of the number of functionally annotated genes based on different public databases.

We identified repeat sequences in the *T*. *bimaculatus* genome with a combination of homology-based and *de novo* approaches using previously established protocol^14^. For the homology-based approach, we used Tandem Repeats Finder^15^ (version 4.04) to detect tandem repeats and used RepeatModeler^16^ (version 3.2.9), LTR_FINDER^17^ (version 1.0.2) and RepeatScout^18^ (version 1.0.2) synchronously to detect repeat sequences in the *T*. *bimaculatus* genome. Combined with Repbase^19^ (Release 19.06), a repeat sequence library was constructed with these results using USEARCH^20^ (version 10.0.240). Then, we used RepeatMasker^16^ (version 3.2.9) to annotate repeat elements based on this library. In another approach, we utilized Repbase^19^ and a Perl script included in the RepeatProteinMasker (submodule in Repeatmasker) program with default parameters to detect TE proteins in the *T*. *bimaculatus* genome. Finally, after removing redundancies, we combined all the results generated by these methods, and a total of 109.92 Mb (27.2% in the *T*. *bimaculatus* genome) sequences were identified as repeat elements (Table 4). Among these repeat elements, long interspersed nuclear elements (LINEs) were the main type, accounting for 12.31% (49.76 Mb). In addition, regarding other repeat elements, there were 24.46 Mb (6.05%) of DNA transposons, 1.19 Mb (0.29%) of short interspersed nuclear elements (SINEs) and 31.55 Mb (7.8%) of long terminal repeats (LTRs) (Figs 2a and 3a Table 4).

**Table 4:** Classification of repeat elements and ncRNAs in *T*. *bimaculatus* genome.

Repeat type		Denove + Repbase Length (bp)	TE protein Length (bp)	Combined TEs length (bp)	Proportion in Genome (%)
DNA		21,029,049	3,437,660	24,459,756	6.05
LINE		37,262,756	12,547,875	49,755,614	12.31
SINE		1,189,529	0	1,189,529	0.29
LTR		25,586,059	5,992,977	31,547,035	7.80
Simple Repeat		8,473,364	0	8,473,364	2.10
Unknow		4,719,800	0	4,719,800	1.17
Total		88,122,922	21,916,443	109,924,780	27.20
**ncRNA type**		**Copy**	**Average Length (bp)**	**Total Length (bp)**	**Propration in Genome (%)**
miRNA		1666	91.11	151786	0.037551
tRNA		753	75.20	56629	0.01401
rRNA	18S	464	113.37	52604	0.013014
	28S	1	121	121	0.00003
	5.8S	9	142.78	1,285	0.000318
	5S	0	0	0	0
	Subtotal	454	112.77	51,198	0.012666
sRNA	CD-box	588	141.15	82,996	0.020533
	HACA-box	84	92.52	7,772	0.001923
	Splicing	77	162.88	12,542	0.003103
	Subtotal	413	144.85	59,821	0.0148

Note: “Denovo” represented the *de novo* identified transposable elements using RepeatMasker, RepeatModeler, RepeatScout, and LTR_FINDER. “TE protein” meant the homologous of transposable elements in Repbase identified with RepeatProteinMask. While “Combined TEs” referred to the combined result of transposable elements identified in the two ways. “Unknown” represented transposable elements could not be classified by RepeatMasker.

For gene structure prediction, we used both homology-based and *de novo* strategies to predict genes in the *T*. *bimaculatus* genome. For homology-based prediction, we mapped the protein sequences of *Oryzias latipes*^21^, *Gasterosteus aculeatus*^22^, *Tetraodon nigroviridis*^23^, *Takifugu rubripes*^24^ and *Oreochromis niloticus*^25^ onto the generated assembly using BLAT^26^ (version 35) with an e-value ≤ 1e-5. Then, we used GeneWise^27^ (version 2.2.0) to align the homologous in the *T*. *bimaculatus* genome against the other five teleosts for gene structure prediction. In the *de novo* approach, we used several software packages, including Augustus^28^ (version 2.5.5), GlimmerHMM^29^ (version 3.0.1), SNAP^30^ (version 1.0), Geneid^31^ (version 1.4.4) and GenScan^32^ (version 1.0). In addition, we also used RNA-seq data (NCBI accession number: SRX5099972) to predict the structure of transcribed genes using TopHat^33^ (version 1.2) and Cufflinks^34^ (version 2.2.1). Using EvidenceModeler^35^ (version 1.1.0), we combined the set of predicted genes generated from the three approaches into a non-redundant gene set and then used PASA^36^(version 2.0.2) to annotate the gene structures. Finally, a total of 21,117 protein-coding genes were predicted and annotated, with an average exon number of 9.71 and an average CDS length of 1573.89 bp in each gene(Fig. 3b and Table 5). For the annotation of candidate non-coding RNA (ncRNA), we used BLASTN^37^ to align the *T*. *bimaculatus* genome against the Rfam database^38^ (version 12.0). As a result, we annotated 1,666 miRNA, 753 tRNA, 928 rRNA and 1162 snRNA genes (Fig. 2a and Table 4).

**Table 5:** Gene structure and function annotation in *T*. *bimaculatus* genome.

**Gene structure Annotation**	
Number of protein-coding gene	21,117
Number of unannotated gene	19
Average transcript length (bp)	7,914.81
Average exons per gene	9.71
Average exon length (bp)	162.13
Average CDS length (bp)	1,573.89
Average intron length (bp)	728.2
**Gene function Annotation**	
	Number (Percent)
Swissprot	20,086 (95.10%)
Nr	20,817 (98.60%)
KEGG	18,307 (86.70%)
InterPro	21,090 (99.90%)
GO	19,934 (94.40%)
Pfam	18,050 (85.50%)
Annotated	21,098 (99.90%)
Unannotated	19 (0.10%)

For gene function annotation, we used BLASTP to align the candidate sequences to the NCBI and Swissport protein databases with E values < 1 × 10^−5^. Then, we performed the functional classification of GO categories with the InterProScan program^39^ (version 5.26) and used KEGG Automatic Annotation Server (KAAS)^40^ to conduct the KEGG pathway annotation analysis. A total of 21,098 genes were successfully annotated, accounting for 99.9% of all predicted genes (Figs 2a, 3c and Table 5).

## Data Records

The raw sequencing reads of all libraries are available from NCBI via the accession numbers SRR8285219- SRR8285227^41^. The assembled genome and sequence annotations are available in NCBI with the accession number SWLE00000000 via the project PRJNA508537^42^.

## Technical Validation

### Evaluating the completeness of the genome assembly and annotation

The final assembly contains 404.41 Mb with a scaffold N50 size of 16.79 Mb (Table 2). Assembly completeness and accuracy were evaluated by multiple methods. First, reads from the short-insert library were re-mapped onto the assembled genome using BWA^12^ (version 0.7.17). A total of 96.97% of the reads mapped to a reference sequence in the genome (98.71% coverage), demonstrating a high assembly accuracy (Table S3). We used Genome Analysis Toolkit^43^ (GATK) (version 4.0.2.1) to identify a total of 1,115.45 SNPs throughout the whole genome, including 1,110.69 K heterozygous SNPs and 4,765 homozygous SNPs (Table S4). In addition, the accuracy of the assembly was verified by the extremely low proportion of homozygous SNPs (1.22 × 10^−5^%) (Table S4).

Assembly completeness was evaluated using Core Eukaryotic Genes Mapping Approach (CEGMA) software^44^ (version 2.3), and a total of 235 core Eukaryotic Genes (CEGs) from the complete set of 248 CEGs (94.67%) were identified in the assembled genome, suggesting the draft genome of *T*. *bimaculatus* was high complete (Table S4). Finally, Benchmarking Universal Single-Copy Orthologues (BUSCO) software^45^ (version 1.22) was used to evaluate the completeness of the assembly with the actinopterygii_odb9 database. A total of 4,254 out of the 4,584 searched BUSCO groups (92.8%) had been completely assembled in our draft genome, suggesting a high level of completeness of the *de novo* assembly (Table S3).

To verify the accuracy of the contig arrangement in 22 chromosomes, we aligned 7,443 (count) 1 K bp small fragments with 50 K bp spacing as anchors of the assembled genome against the published *T*. *rubripes* genome (FUGU5)^24,46^ to compare consistency between these two genomes. The 22 chromosomes we identified in the *T*. *bimaculatus* genome aligned exactly against the chromosomes of the *T*. *rubripes*, suggesting high continuity with the *T*. *rubripes* genome (Fig. 2b).

The predicted gene models we used were integrated by EvidenceModeler, and a total of 18,706 genes were predicted by all three gene structure prediction strategies, which representing 88.58% of the 21,117 predicted genes (Fig. 3b). Notably, this validation procedure is limited by the gene expression in the mixture of tissues used for RNA-Seq. Therefore, considering that transcriptomic data derived from different tissues will cover distinct sets of expressed genes, it is conceivable that more genes could be validated.

### Gene family identification and phylogenetic analysis of *T*. *bimaculatus*

To identify gene families among *T*. *bimaculatus* and other species, we download the protein sequence of *Branchiostoma belcheri*^47^(outgroup), *Ciona intestinalis*^48^ (outgroup), *Danio rerio*^49^, *Gadus morhua*^50^, *Gasterosteus aculeatus*^22^, *Latimeria chalumnae*^51^, *Lepisoteus oculatus*^52^, *Mola mola*^53^, *Oryzias latipes*^21^, *Oreochromis niloticus*^25^, *Takifugu rubripes*^24^ and *Tetraodon nigroviridis*^23^. We removed those protein sequences shorter than 30 amino acids in the proteome set of the above thirteen species and used OrthoMCL^54^ to construct gene families. A total of 20,741 OrthoMCL families were built using the previously all-to-all BLASTP strategy^55^.

**Fig. 4:**
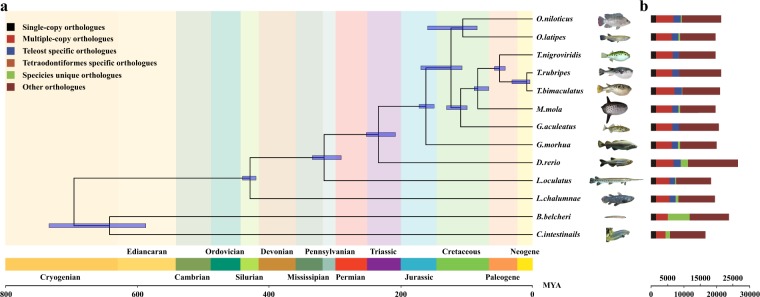
Divergence times and distribution of different types of orthologues in representative species. (**a**)Estimated divergence times of representative species based on the phylogenomic analysis. The blue bars in the ancestral nodes indicate the 95% confidence intervals of the estimated divergence time (MYA, million years). Different background colours represent the corresponding geological age. (**b**) Distribution of different types of orthologues in the selected representative species.

To reveal the phylogenetic relationships among *T*. *bimaculatus* and other species, we identified 1,479 single copy ortholog families from the 13 species (as described above) (Table S5) and aligned the protein sequences of these 1,497 orthologues using MUSCLE (version 3.8.31)^56^. Then we used Gblocks^57^ to extract the well-aligned regions of each gene family alignment and converted protein alignments to the corresponding coding DNA sequence alignments using an in-house script. For each species, we combined all translated coding DNA sequences to a “supergene”. Finally, we used RAxML (version 8.2.12)^58^ with 500 bootstrap replicates to generate trees. Using molecular clock data from the TimeTree database^59^, MCMCTREE (PAML package)^60^ were employed to estimate the divergence time based on the approximate likelihood calculation method. The phylogenetic relationships among the other fish species were consistent with several previous studies^8,14,61^. Based on the phylogenetic analysis, we inferred that *T*. *bimaculatus* speciated approximately 9.1 million years ago from the common ancestor of Takifugu (Fig. 4).

## Supplementary Information

Supplementary Materials

## Data Availability

The versions, settings and parameters of the software used in this work are as follows: Genome assembly: (**1) Falcon:** version 1.8.2; all parameters were set as default; (**2) Quiver:** version: 2.1.0; parameters: all parameters were set as default; (**3) pilon**: version:1.22; all parameters were set as default; (**4) LACHESIS**: parameters: RE_SITE_SEQ = AAGCTT, USE_REFERENCE = 0, DO_CLUSTERING = 1, DO_ORDERING = 1, DO_REPORTING = 1, CLUSTER_N = 24, CLUSTER_MIN_RE_SITES = 300, CLUSTER_MAX_LINK_DENSITY = 4, CLUSTER_NONINFORMATIVE_RATIO = 10, REPORT_EXCLUDED_GROUPS = −1; Genome annotation: (**1) RepeatProteinMask**: parameters: -noLowSimple -pvalue 0.0001 -engine wublast. (**2) RepeatMasker**: version: open-4.0.7; parameters: -a -nolow -no_is -norna -parallel 1. **(3) LTR_FINDER**: version:1.05; parameters: -C -w 2. (**4) RepeatModeler**: version: open-1.0.10; parameters:-database genome -engine ncbi -pa 15. (**5) RepeatScout**: version: 1.0.5; parameters: all parameters were set as default. (**6) TRF**: matching weight = 2, mismatching penalty = 7, INDEL penalty = 7, match probability = 80, INDEL probability = 10, minimum alignment score to report = 50, maximum period size to report = 2000, -d –h. (**7) Augustus**: version:3.1.2; parameters:–extrinsicCfgFile–uniqueGeneId = true–noInFrameStop = true–gff3 = on–genemodel = complete–strand = both. (**8) GlimmerHMM**: version:3.0.3; parameters: -f –g. (**9) Genscan**: -cds. (**10) Geneid**: version: 1.2; parameters: -P -v -G -p geneid. (**11) Genewise**: version: 2.4.0; parameters: -trev -genesf -gff –sum. (**12) BLAST**: version 2.7.1; parameters: -p tblastn -e 1e-05 -F T -m 8 -d. **(13) EVidenceModeler**: version: 1.1.1; parameters: G genome.fa -g denovo.gff3 –w weight_file -e transcript.gff3 -p protein.gff3–min_intron_length 20. (**14) PASA**: version: 2.3.3; parameters: all parameters were set as default. Gene family identification and phylogenetic analysis: (**1) Blastp:** parameters: -e 1e-7 -outfmt 6. (**2) Orthomcl:** parameters: all parameters were set as default. (**3) MUSCLE:** version 3.8.31; parameters: all parameters were set as default. (**4) Gblocks:** version: 0.91b; parameters: all parameters were set as default. (**5) RAxML:** version: 8.2.12; parameters: -n sp -m PROTGAMMAAUTO -T 20 -f a. (**6) MCMCTREE:** parameters: all parameters were set as default.
