# Supplementary material for: The sequence and de novo assembly of Takifugu bimaculatus genome using PacBio and Hi-C technologies
Source: Sci Data. 2019 Sep 30;6:187. doi: 10.1038/s41597-019-0195-2 (PMC6768875; doi:10.1038/s41597-019-0195-2)
Supplement: Supplementary file 1 — Supplementary Materials [file 41597_2019_195_MOESM1_ESM.pdf]

Supplementary contents	Page Number
**Fig S1**	2
**Fig S2**	3
**Table S1**	4
**Table S2**	5
**Table S3**	6
**Table S4**	7
**Table S5**	8

Supplementary Figure

Figure S1. (A) A picture was showing about T. bimaculatus; (B) the location of sample collection.

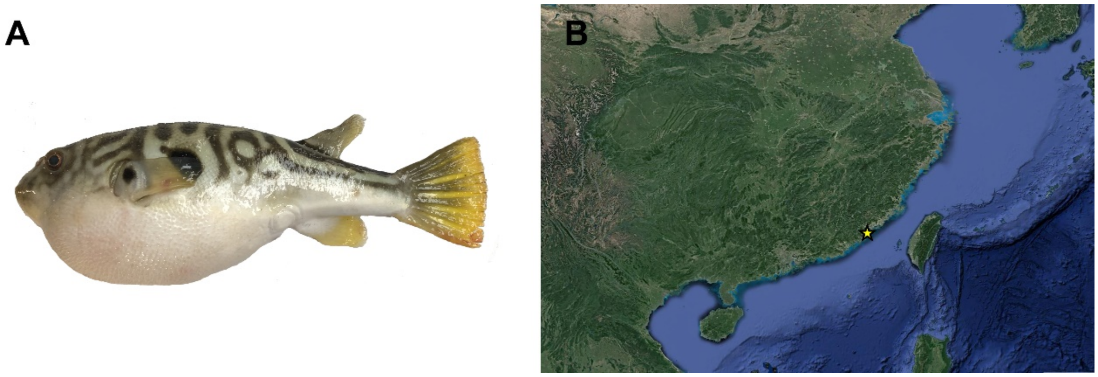

Figure S2. Distribution profiles of 17 K-mer counts in Illumina sequence reads.

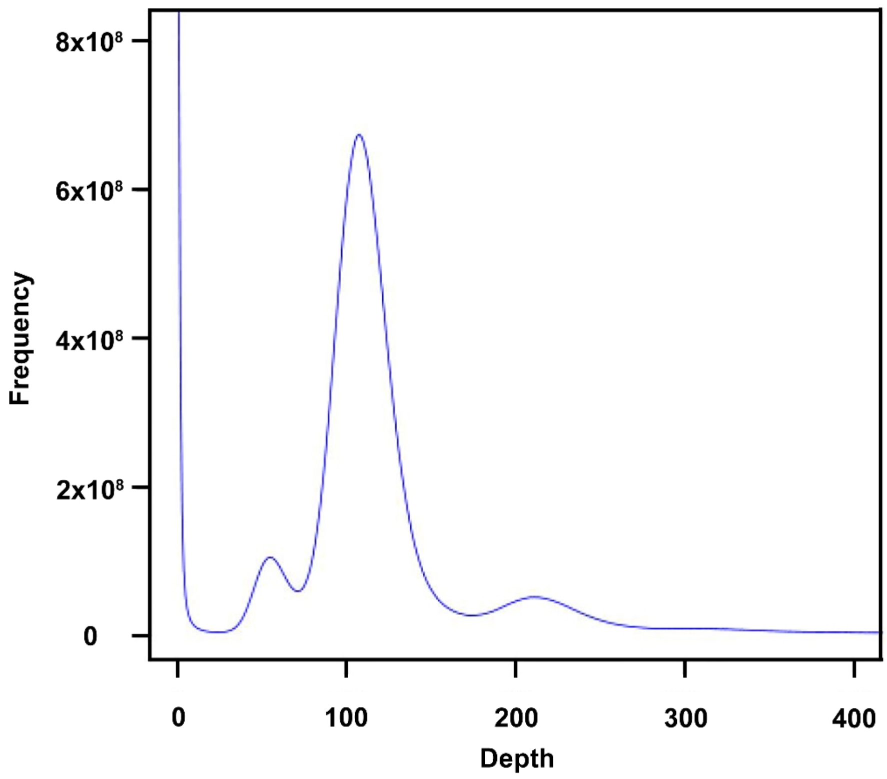

Supplementary Table

Table S1. List of the tissues used in RNA-seq.

Tissue	BioSample	BioProject
Eye	SAMN10524748	PRJNA508537
Brain	SAMN10524748	PRJNA508537
Heart	SAMN10524748	PRJNA508537
Liver	SAMN10524748	PRJNA508537
Intestine	SAMN10524748	PRJNA508537
Gill	SAMN10524748	PRJNA508537
Skin	SAMN10524748	PRJNA508537
Spleen	SAMN10524748	PRJNA508537
Kidney	SAMN10524748	PRJNA508537

Table S2. Genome statistics based on 17 K-mer analysis.

K-mer	K-mer number	K-mer Depth	Genome Size (Mbp)	Revised Genome Size (Mbp)	Heterozygous Ratio (%)	Repeat (%)
17	49,973,744,256	107	401.62	393.15	0.55	25.29

*Genome size: estimated genome size; Revised Genome size: revised genome size that eliminated the influence caused by error kmer counts

Table S3. The genome coverage statistics of Illumina sequence reads.

	Percentage
Mapping rate (%)	96.97%
Coverage (%)	98.71%
Coverage at least 4X (%)	98.10%
Coverage at least 10X (%)	97.41%
Coverage at least 20X (%)	96.54%

Table S4. Details of accuracy and completeness validation of genome assembly.

Illumina Reads Mapping	Number	Percentage
All SNP	1,115,451	0.00286
Heterozygous SNP	1,110,686	0.00285
Homology SNP	4,765	$1.22X10^{-5}$
CEGMA		
Number of completely assembled CEGs	235	
Proportion of completely assembled CEGs (%)	94.76	
Number of assembled CEGs	237	
Proportion of assembled CEGs (%)	95.56	
BUSCO	Number	Percentage
All BUSCOs used	4584	-
Complete BUSCOs	4254	0.928
Fragmented BUSCOs	160	0.035
Missing BUSCOs	170	0.037

Table S5. The statistics of different types orthologues in representative species.

Species	Total	SC*	MC*	Teleost Specific	Species Unique	Ts*	Other
Bra	23868	1479	3660	-	6653	-	12076
Cin	16671	1479	2860	-	1504	-	10828
Coe	19569	1479	4195	1846	815	-	11234
Dre	26586	1479	5322	2227	2200	-	15358
Gac	20787	1479	5014	1987	116	-	12191
Gam	20095	1479	4767	1937	600	-	11312
Leo	18341	1479	4073	1790	266	-	10733
Ola	19765	1479	4779	2001	523	-	10983
Orn	21437	1479	5259	2208	377	-	12114
Mam	19605	1479	4988	1963	349	19	10807
Tbi	21117	1479	5716	2133	158	15	11616
Tni	19640	1479	5096	2009	0	15	11041
Tru	21317	1479	5015	1988	81	15	12739

*SC: single copy orthologues; MC: Multiple copy orthologues; Ts: Tetraodontiformes specific orthologues.
